# Government intervention, internal control, and technology innovation of SMEs in China

**DOI:** 10.3389/fpsyg.2022.960025

**Published:** 2022-08-01

**Authors:** Sun Ye, Sun Yi, Shao Fangjing, Qi Yuzhu

**Affiliations:** ^1^Business and Management School, Jilin University, Changchun, China; ^2^College of Accounting, Jilin University of Finance and Economics, Changchun, China; ^3^School of Economics, Jilin University, Changchun, China

**Keywords:** government intervention, internal control quality, enterprise technology innovation, China, small and medium sized enterprises (SMEs)

## Abstract

Under the innovation-driven development strategy, the improvement of the core competitiveness of enterprises demonstrates increasing dependence on the ability of technological innovation. In this article, data of A-share listed companies in Shanghai and Shenzhen stock markets from 2008 to 2018 were selected as research samples for the analysis of the influencing factors and mechanism of enterprise technological innovation from the dual perspectives of the external economic environment and internal management system based on the use of the fixed-effect model. The results show that government intervention significantly hinders enterprises' investment in resources for technological innovation, and less government intervention can improve the innovation investment of enterprises. The intervention of internal control fails to bring institutional advantages; rather, it aggravates the negative effect of government intervention on enterprise technological innovation. The research enriches the existing academic research results on government intervention, internal control quality, and enterprise technological innovation. The findings provide experience for accelerating the marketization process in China, enterprise governance, and improving the level of enterprise technological innovation.

## Preamble

Innovation is the cornerstone of national development and the source of strength for national rejuvenation (Hong et al., 2016). General Secretary Xi Jinping made it clear in his report to the 19th National Congress of the Communist Party of China that “we must be equipped with strong capacity in scientific and technological strength and innovation to realize the Chinese dream for national rejuvenation.” In recent years, the number of enterprises engaged in technological innovation has increased from 6.5% in 2008 to 28% in 2018, with an average annual growth rate of 1.95%. However, among enterprises carrying out R&D activities, the overall investment is at a low level, and there are significant differences in the attitude of enterprises toward technological innovation activities (Cao et al., 2019).

The research and development investment of China's A-share listed companies accounts for 19.29% of the maximum revenue, but not more than 20%, while the minimum is only 0.0127%. In order to alleviate this problem, China has issued a series of policies, laws, and regulations to encourage enterprises to carry out innovative activities through government intervention (Li et al., 2019). However, some scholars put forward the opposite view that the market-based competition mechanism would enable enterprises to improve their competitiveness. In light of input-output inequality caused by the imitability of R&D, the higher the degree of marketization, the more improved the law, and the more efforts in the protection of R&D results will stimulate more enterprises to invest in R&D to improve their core competitiveness (Xiaoli, 2011).

Since the outbreak of the Enron incident, internal control has gradually received universal attention and recognition worldwide. Internal control is endowed with the characteristics of heterogeneous resources that fail to be mimicked. As a result, it can reduce the uncertainty of income and cost in technological innovation activities and promote enterprises to increase investment in technological innovation (Deng et al., 2019). However, other scholars believe managers would prefer risk aversion with the adoption of internal control, and it is difficult to motivate employees for innovation, thus seriously hindering the development of technological innovation in an enterprise (Zhu et al., 2018; Jia et al., 2021). The scholarly opinion is currently not unified, and its influence mechanism on enterprise technological innovation still needs further exploration.

According to the above mentioned, this article examines the relationship between a market economy and enterprise technological innovation under government intervention. It examines the moderating role of internal control quality in it. It is conducive to a more systematic understanding of the impact of government intervention and internal control quality on enterprise technological innovation in the enterprise transformation and upgrading process, which has vital theoretical and practical significance.

## Literature review

### Government intervention and technological innovation of enterprises

As technological innovation activities possess the characteristics of external effect and public goods, the effect of enterprise technological innovation is subject to the influence of market conditions. Therefore, the government plays an essential role in promoting the technological innovation of enterprises (Lin and Luan, 2020). There are still differences in the relationship between government intervention and enterprises based technological innovation, which can be divided into the school of facilitation and the school of inhibition theory (Luan et al., 2020).

Scholars of the facilitation school believe that government intervention, as a positive signal, motivates enterprises to carry out R&D activities (Guo et al., 2018). Government intervention in enterprises' R&D activities is mainly supported by formulating various fiscal and financial policies and adopting policy tools such as government subsidies and tax incentives (Yang et al., 2019). However, scholars of the inhibitory school found that some conducts of governments deprived enterprises of autonomy in technology development. Liu and Liu (2007) believed that reducing government intervention would improve the company's R&D investment.

Lingling (2018) pointed out that in poor areas in the external economic environment, the governments were more inclined to intervene in enterprise management. Consequently, enterprises fail to obtain high-quality resources through regular market competition. Hence, enterprises, driven by pressure, must choose informal methods such as establishing political connections and rent-seeking to obtain government protection, resulting in a tremendous waste of resources (Zhao et al., 2021). Then how does government intervention influence enterprises' technological innovation, and does it have a positive or negative effect on enterprise technological innovation? As for the influence mechanism of government intervention on enterprise technological innovation, the academia failed to reach a consensus, which needs further research and discussion.

### Internal control quality and technology innovation of enterprises

With the existence of information asymmetry between agent and consignor in the modern corporate system, the principal-agent problem has significantly impacted corporate governance (Eisenhardt, 1989; Lan and Heracleous, 2010). Studies have discovered that internal control plays a significant role in promoting enterprises' technological innovation ability, and investment in enterprises' innovation increases with the improvement of internal control (Vasilev et al., 2017). Among enterprises with an effective internal control system, the self-interested behavior of enterprise managers is often restricted, and the information asymmetry and principal-agent problems in the investment operation of enterprises' R&D activities will be effectively alleviated (Wang et al., 2022).

The fundamental internal control lies in institutional constraint, which inevitably conflicts with the flexibility of technological innovation (Lovelace et al., 2001). In his study, Zhang (2007) found that reasonable internal control would make enterprise managers more vulnerable to risks, thus leading to less invisible income. Ultimately, managers' severe lack of innovation motivation is developed. Bargeron et al. (2010) believes that SOX inhibits enterprises' R&D activities, and strict internal control is bound to reduce the chance of speculation of enterprise top managers, who are unwilling to bear the consequences of enterprise R&D failure, which seriously retards the improvement of enterprises' innovation ability.

Through sorting out and summarizing relevant academic literature, it is found that there are significant research achievements on government intervention and enterprises based technological innovation in the existing literature. However, little literature introduces internal control quality to explore its moderating effect on the relationship between government intervention and enterprises-based technological innovation (Li, 2020). Based on this, research on the influence mechanism and effect of government intervention, internal control quality, and enterprise technological innovation provides a valuable perspective for guiding enterprises to strengthen technological innovation and improve the upgrading of industrial structure (Shen et al., 2020).

## Theoretical analysis and research-based hypothesis

Based on the current governmental system in China, this part of the content carries out a theoretical analysis of the relationship between government intervention, enterprise internal control quality, and enterprises based technological innovation. It puts forward research hypotheses on this basis.

### Analysis of the impact of government intervention on enterprises based on technological innovation

The primary purpose of the government to provide direct financial resource subsidies to enterprises is to promote and stimulate enterprises to increase investment in R&D and guide technological innovation to tackle critical problems as the market failure occurs to promote enterprises to improve the development level of technological innovation and adjust industrial structure (Nam and An, 2017; Lee et al., 2020). Since the last century, the “promotion tournament mode” has had a strong incentive effect on government officials who care about their official careers to make their political promotion goal compatible with the economic growth goal of their jurisdiction (Chen et al., 2016).

However, on most occasions, the long duration of enterprises in technological innovation activities makes it difficult for managers to produce economic benefits during their term of office, which seriously affects the political performance of local government officials during the term of office (Liu and Luo, 2019). Former officials rose to the challenge of motivating enterprises to carry out technical innovation. However, later officials showed more preference for directly taking the innovative results achieved by the former officials. The innovation of “One sow and another reaps” seriously affected the fairness of performance appraisal, leading to “short-sighted” regional economic growth (Liu et al., 2016).

Therefore, technological innovation activities with a long cycle are not encouraged, and it hinders the technological innovation activities of enterprises. As a result, government officials tend to make “short-sighted” economic decisions during their tenure, neglecting projects such as technological innovation that bring long-term economic benefits (Ganda, 2019). Moreover, enterprises with more government intervention tend to obtain resources through political means such as rent-seeking and political connections rather than technological innovation (Liu and Xie, 2020). Therefore, the following hypotheses are proposed by us:

**H1:** Government intervention inhibits technological innovation of enterprises.

### Analysis of the impact of internal control quality on enterprises based on technological innovation

Due to the asymmetry of information, the principal fails to obtain the agent's behavior in real-time (Zhong et al., 2022). At this time, the agent will likely conduct self-interested behavior and damage the principal's interests. With high-quality internal control, executives' behavior is often constrained (Li M. et al., 2021). Compared with other daily production and operation activities, technological innovation requires enterprise managers to break the stereotype and develop new products or businesses. Internal control, as a system to regulate enterprise behavior, can reduce enterprise production and operation risks through clear division of labor, separation of responsibilities, standard authorization, and approval (Chang et al., 2020). However, the too strict internal control system will make managers in technological innovation by the constraints of procedural approval and forced to shelve innovation projects.

Moreover, limited by the risks of innovation activities, the management will likely bear the corresponding responsibilities and consequences once the innovation fails. Their enthusiasm for innovation is significantly weakened, so they choose conservative management (Zeng et al., 2020). Employees are also unable to turn innovative ideas into innovative results.

In conclusion, managers, on most occasions, will be required to be responsible for decision-making behaviors based on the internal control system. Hence, managers usually avoid high-risk projects to avoid the occurrence of damaging personal interests, which seriously affect the technological innovation of enterprises (Wu et al., 2022). The standardized internal control system will also bring employees a strong sense of bondage and reduce their enthusiasm for innovation. Therefore, we propose the following hypothesis:

**H2:** The quality of internal control inhibits the technological innovation of enterprises.

### Analysis of the impact of internal control quality on the relationship between government intervention and enterprise technological innovation

Enterprises with more government intervention enjoy the advantages in resources brought by the government, and the enterprises often lack effective incentives to strengthen corporate management (Chen et al., 2011). Some local governments interfere in equity shares by acting as shareholders of state-owned enterprises, thus affecting enterprises' principal-agent relationship (Salmenkaita and Salo, 2002). In the case of relatively stringent internal control, the enterprises are required to solve the external negative influence of government intervention on the enterprise. Besides, enterprises will face more internal institutional constraints. The double restraints put senior managers and enterprise employees in strict and standardized working mode (Balakrishnan et al., 2010).

The inability to effectively transform innovation into innovation achievements further aggravates the restraining effect of government intervention on technological innovation and reduces the level of technological innovation of enterprises (Zhang et al., 2014). However, flexible internal control can ease the fear of senior managers on innovation risks and consequences of innovation failure; technological innovation can be carried out without government support to alleviate the negative impact of government intervention on enterprise technological innovation (Wang et al., 2018).

To sum up, different quality of internal control will have different degrees of influence on the relationship between government intervention and enterprise technological innovation: when the quality of internal control is high, government intervention has a higher degree of inhibition on enterprise technological innovation (Gao et al., 2021); On the contrary, with low internal control quality, government intervention has a low degree of inhibition on enterprise technological innovation (Li G. et al., 2021). Therefore, we propose the following hypothesis:

**H3:** Internal control quality enhances government intervention's restraining effect on enterprise technological innovation.

### Research model

**Figure 1:**
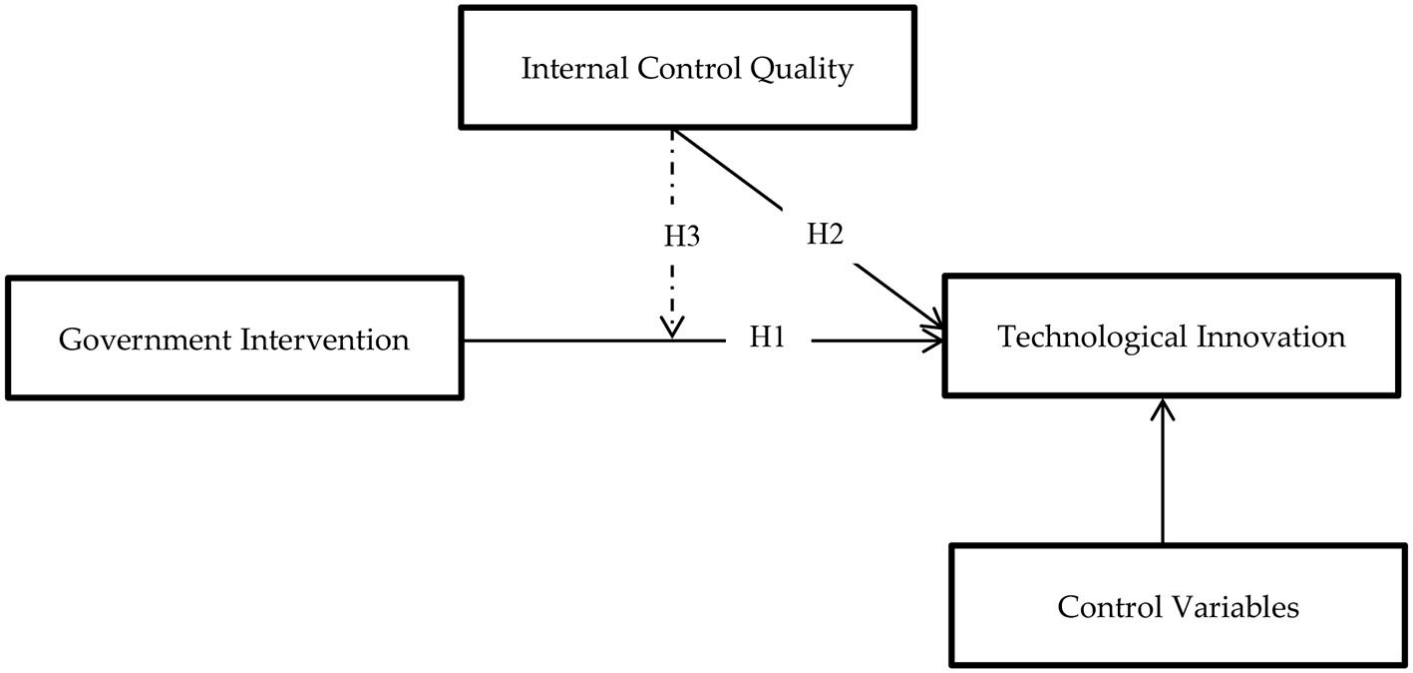
Theoretical model.

Figure 1 shows the theoretical model regarding government intervention and technological innovation.

## Research design

### Sample selection and data sources

This article selected A-share listed companies in Shanghai and Shenzhen stock markets from 2008 to 2018 as the initial research samples. In order to ensure the validity of data, the samples are processed as follows: (1) The samples of the financial industry are excluded; (2) ST and ^*^ ST enterprise samples are removed; (3) Enterprise samples with missing data are eliminated. A total of 13,159 observations were obtained from 2,307 enterprises. Data on government intervention are derived from Wang et al.'s Report on Marketization Index by Provinces in China (2018). The interpolation method is adopted to supplement the missing values of individual provinces. Data related to the quality of internal control comes from “DIB· Internal Control Index of Chinese Listed Companies,” and other financial data are derived from or calculated from the CSMAR series research database of China. EXCEL and Stata13 are used in this article to sort out and analyze sample data and conduct empirical research. In this article, a 1% tail reduction is adopted.

### Variable design

#### Explained variables

Technology Innovation (RD) of enterprises. Research and development expenditure, number of patent applications, and sales of new products are mainly used to measure the quality of technological innovation. Not all the inventions can apply for a patent, technology innovation and patent application show a strong hysteresis, and new product sales database data is derived from China industrial enterprises. Due to different statistical caliber, the data presented low coherence, which fails to meet this research paper's needs. Therefore, this article selected R&D investment intensity (RD) as an indicator to measure the technological innovation of enterprises. Meanwhile, considering the influence of different enterprise sizes, the proportion of R&D expenditure in operating revenue was selected as the research indicator.

#### Explanatory variables

##### Government intervention (Market)

The market index measured the degree of government intervention in The Report of Marketization Index by Provinces in China compiled by Wang et al. The index level is directly proportional to the degree of marketization and inversely proportional to the degree of government intervention. Therefore, the degree of government intervention is negatively correlated with the degree of regional marketization.

#### Moderating variables

##### Internal control quality (IC)

This article uses DIB internal control Index (IC), which is widely recognized and commonly used in academia, as a measurement index. This index integrates the current situation of domestic listed companies implementing an internal control system based on the realization degree of internal control objectives. At the same time, defects in internal control are modified as a correction variable to finally form an internal control index that comprehensively reflects the internal control level and risk control ability of listed companies.

#### Control variables

Based on existing studies, this article selects enterprise Size (Size), asset-liability ratio (Lev), return on asset (ROA), enterprise growth, ownership concentration (Fhold), and enterprise age (Age) as control variables. At the same time, this article sets the annual dummy variable (Year) and Industry dummy variable (Industry) to eliminate the influence of fixed effect.

The definitions of all variables studied in this article are shown in Table 1.

**Table 1:** Definition and description of study variables.

**The variable name**		**Symbol**	**Definition**
Variable being explained	R and D investment intensity	RD	(R and D investment of enterprises/Operating revenue) * 100 (Percentage sign is omitted for convenience of data display)
Explanatory variable	Government intervention	Market	Marketization Index in Report on Market Index by Province in China (2016)
Moderating variables	Internal quality control	IC	DIB Internal Control Index /100 (Percentage sign is omitted for convenience of data display)
Control variable	Enterprise-scale	Size	The natural log of the total assets of the firm
	Asset-liability ratio	Lev	Total liabilities/total assets at the end of the period * 100 (Percentage sign is omitted for convenience of data display)
	Profitability	ROA	The year-end return rate on Total assets * 100 (Percentage sign is omitted for convenience of data display)
	Enterprise growth	Growth	The growth rate of business revenue * 100 (Percentage sign is omitted for convenience of data display)
	Equity concentration	Fhold	Share-holding proportion of the largest shareholder
	Enterprise age	Age	The difference between the current year and the year of establishment of the enterprise
	Annual basis	Year	Annual pseudo-variable
	Industry	Industry	Industry pseudo-variable, according to Guidelines on Industry Classification of Listed Companies of China Securities Regulatory Commission (CSRC) (2012 Edition)

#### Construction of the model

According to the results of the Hausman test on panel data, the fixed effects model is adopted in this article. Based on the research content of this article, that is, the relationship between government intervention, internal control quality, and enterprise technological innovation, a multiple regression model is established for empirical analysis to verify the hypotheses proposed above.

In order to test the influence of government intervention on enterprises based on technological innovation in Hypothesis 1, the model is established as follows:


(1) $$\begin{array}{l} \begin{array}{c} RD_{i,t}=\alpha_{0}+\alpha_{1}Market_{i,t}+\alpha_{2}Controls+\sum^{\text{​}}Year \end{array}\\ \;\begin{array}{c} +\sum^{\text{​}}Industry \end{array}\; \end{array}$$

In order to test the influence of internal control quality on enterprises based on technological innovation in Hypothesis 2, the model is established as follows:


(2) $$\begin{array}{l} \begin{array}{c} RD_{i,t}=\beta_{0}+\beta_{1}IC_{i,t}+\beta_{2}Controls+\sum^{\text{​}}Year \end{array}\\ \;\begin{array}{c} +\sum^{\text{​}}Industry \end{array} \end{array}$$

In order to test the regulating mechanism of internal control quality on the impact of government intervention on enterprise technological innovation in Hypothesis 3, the interaction terms of government intervention and internal control variables are cited to establish the model:


(3) $$\begin{array}{l} \begin{array}{c} RD_{i,t}=\gamma_{0}+\gamma_{1}Market_{i,t}+\gamma_{2}IC_{i,t}+\gamma_{3}Market_{i,t}\times IC_{i,t} \end{array}\\ \;\begin{array}{c} +\gamma_{4}Controls+\sum^{\text{​}}Year+\sum^{\text{​}}Industry \end{array} \end{array}$$

## Analysis of empirical results

### Descriptive statistics

Table 2 refers to the descriptive statistics of each variable. As seen in Table 2, the average value of enterprise technological innovation (RD) is 3.221, indicating that the average R&D investment in the operating revenue of Chinese enterprises accounts for only 3.221%, with the standard deviation standing at 3.228 the minimum value of 0.0127. Besides, the maximum value is 19.29, indicating that there are still significant differences in technological innovation among different enterprises in China. The average value of the government intervention index reaches 8.013, the minimum value is 2.98, and the maximum value is 10.49, indicating significant differences in the level of government intervention in different regions of China. The minimum value of the internal control index is 3.68, the maximum value is 8.886, and the average value is 6.716, indicating that most enterprises' internal control quality is high.

**Table 2:** Descriptive statistics.

**Variables**	**Sample capacity**	**Mean value**	**Standard deviation**	**Minimum value**	**Maximum value**
Market	13,159	8.013	1.783	2.980	10.49
IC	13,159	6.716	0.732	3.768	8.866
RD	13,159	3.221	3.228	0.0127	19.29
Size	13,159	22.26	1.274	19.93	26.23
Lev	13,159	43.25	19.40	6.031	86.20
ROA	13,159	5.255	4.527	−0.766	22.31
Growth	13,159	19.59	38.60	−37.99	255.9
Fhold	13,159	35.78	14.95	8.770	74.98
Age	13,159	21.79	5.178	7	53

### Correlation test

According to the Pearson correlation analysis results of primary variables in Table 3, the correlation between government intervention variables and enterprise technological innovation variables is 0.059, which is positively correlated at the significant level of 1%. In other words, enterprises in regions with higher marketization levels enjoy lower government intervention and higher enterprise technological innovation levels, which is consistent with H1. The correlation coefficient between government intervention variables and internal control quality is 0.092, showing a positive relationship of 1% significant level, indicating that the improvement of marketization degree helps enterprises further improve the internal control system to improve the effectiveness of internal control.

**Table 3:** Pearson-based correlation analysis results among main variables.

	**VIF**	**RD**	**Market**	**IC**	**Size**	**Lev**	**ROA**	**Growth**	**Fhold**	**Age**
RD		1								
Market	1.04	0.059^***^	1							
IC	1.19	−0.070^***^	0.092^***^	1						
Size	1.47	−0.206^***^	−0.136^***^	0.207^***^	1					
Lev	1.65	−.316^***^	−0.071^***^	0.042^***^	0.496^***^	1				
ROA	1.39	0.116^***^	0.059^***^	0.285^***^	−0.083^***^	−0.395^***^	1			
Growth	1.06	−0.018^**^	0.000	0.142^***^	0.049^***^	0.053^***^	0.178^***^	1		
Fhold	1.08	−0.135^***^	−0.021^**^	0.134^***^	0.210^***^	0.067^***^	0.073^***^	−0.001	1	
Age	1.05	−0.168^***^	0.020^**^	−0.022^**^	0.074^***^	0.161^***^	−0.074^***^	−0.021^**^	−0.120^***^	1

*, ** and *** signify the significant results at 0.1, 0.05 and 0.01 levels respectively.

The correlation coefficient between the quality of internal control and the technological innovation of enterprises is −0.07 (1% significant level), showing a negative relationship, indicating that the improvement of internal control will reduce the technological innovation level of enterprises consistent with H2 in this article. As for the control variables, the company size, asset-liability ratio, profitability, enterprise growth, ownership concentration, and enterprise age are all significantly correlated with the technological innovation level of enterprises, indicating the rationality of the model established in this article. The correlation coefficients between all variables were <0.5, and the maximum VIF variance inflation factor was 1.65, indicating no severe multicollinearity relationship between all variables.

Table 4 shows the test results of the fixed-effect model for the main hypothesis in this article.

**Table 4:** Regression analysis results of government intervention, internal control quality, and enterprise technological innovation.

	**Model (1)**	**Model (2)**	**Model (3)**
Market	0.132^***^		0.128^***^
	(4.32)		(4.23)
IC		−0.067^**^	−0.079^**^
		(−2.68)	(−3.12)
Market ^*^ IC			−0.076^***^
			(−3.56)
Size	−0.024	−0.018	−0.015
	(−0.43)	(−0.32)	(−0.27)
Lev	−0.022^***^	−0.022^***^	−0.022^***^
	(−8.64)	(−8.68)	(−8.61)
ROA	−0.008	−0.004	−0.004
	(−1.03)	(−0.55)	(−0.58)
Growth	−0.003^***^	−0.003^***^	−0.003^***^
	(−5.88)	(−5.64)	(−5.66)
Fhold	−0.007^*^	−0.007^*^	−0.0069^*^
	(−2.20)	(−2.09)	(−2.13)
Age	−0.050^***^	−0.053^***^	−0.051^***^
	(−5.03)	(−5.22)	(−5.12)
Industry	Control	Control	Control
Year	Control	Control	Control
_cons	1.886	2.893^*^	2.223
	(1.57)	(2.45)	(1.84)
*N*	13,159	13,159	13,159
*R* ^2^	0.145	0.144	0.146

*, **, and *** were significant at 0.1, 0.05 and 0.01 levels respectively. The values in parentheses are t values.

### Regression-based analysis

#### Regression analysis on government intervention and enterprises-based technological innovation

According to model (1) in Table 4, the regression coefficient between government intervention (Market) and enterprises-based technological innovation (RD) is 0.132 (1% significant level). The regression results show that the government intervention variables positively impact enterprises' technological innovation levels. That is, the region with weak government intervention is more conducive to the improvement of the technological innovation level of enterprises. The government provides many political protection and resources for enterprises to ensure their smooth operation. Therefore, enterprises may ignore the long-term benefits brought by technological innovation activities and would preferably seek rent or establish political connections for their option. The regression analysis of model (1) verifies hypothesis H1: increasing government intervention inhibits enterprises' technological innovation activities.

#### Regression analysis on internal control quality and enterprise-based technology innovation

The regression results of model (2) in Table 4 show that the regression coefficient between the quality of internal control (IC) and enterprise technological innovation (RD) is −0.067, which is significant at 1% level, indicating that the strengthening of internal control fails to bring institutional advantages to enterprise technological innovation necessarily. Instead, it restricts enterprise operation and production, weakens the innovation enthusiasm of management and employees, and then reduces the technological innovation level of enterprises. Under the constraints of strict internal control systems, many innovative projects may have to be shelved due to authorization approval. The high risk and uncertainty of innovation activities also affect the management decisions of enterprise managers to a certain extent, and they may avoid innovation because they are unwilling to take innovation risks. The regression results effectively confirm hypothesis H2.

#### Regression analysis on government intervention, internal control quality, and enterprise technological innovation

Model (3) in Table 4 has analyzed the interactive influence of government intervention and internal control quality on enterprise technological innovation. The regression results show that the regression coefficient of the cross-term of government intervention (Market) and internal control quality (IC), and enterprise technological innovation (RD) is −0.076, and the *T* value is −3.56, which is significant at a 1% level. The regression results show that internal control quality has a negative moderating effect on the marketization index and government intervention index on technological innovation, which is contrary to the positive influence of the marketization index and enterprise technological innovation in Model 1, demonstrating that the improvement of internal control quality can inhibit the promotion effect of marketization on enterprise technological innovation. Since the degree of government intervention is negatively correlated with the degree of marketization, the regression conclusion is that the improvement of internal control can strengthen the inhibition effect of government intervention on enterprise technological innovation. The regression results effectively confirmed hypothesis H3.

### Robustness test

#### Replacing the research samples with data from high-tech enterprises

The research samples of this article are A-share listed companies in Shanghai and Shenzhen. We adopt the commonly used research methods in academia and utilize the total scores for marketization in the Report on Market Index by Province in China as the measure of government intervention. However, to test the model's robustness, the sample data of high-tech enterprises were selected for further regression tests. As shown in Table 5, the results were consistent with the initial results, verifying the conclusion's validity.

**Table 5:** Regression results of samples from high-tech enterprises.

	**Model (1)**	**Model (2)**	**Model (3)**
Market	0.118^***^		0.116^***^
	−4.04		−4.01
IC		−0.061^*^	−0.072^**^
		(−2.47)	(−2.88)
Market ^*^ IC			−0.058^**^
			(−2.74)
Size	−0.039	−0.033	−0.03
	(−0.71)	(−0.60)	(−0.55)
Lev	−0.019^***^	−0.020^***^	−0.019^***^
	(−8.20)	(−8.23)	(−8.18)
ROA	−0.001	0.002	0.002
	(−0.17)	−0.23	−0.23
Growth	−0.003^***^	−0.003^***^	−0.003^***^
	(−5.60)	(−5.37)	(−5.39)
Fhold	−0.007^*^	−0.007^*^	−0.007^*^
	(−2.25)	(−2.12)	(−2.20)
Age	−0.051^***^	−0.053^***^	−0.052^***^
	(−5.47)	(−5.64)	(−5.56)
Industry	Control	Control	Control
Year	Control	Control	Control
_cons	2.155	3.043^**^	2.440^*^
	−1.83	−2.63	−2.06
*N*	12,178	12,178	12,178
*R* ^2^	0.157	0.157	0.158

*, **, and *** were significant at 0.1, 0.05 and 0.01 levels respectively. The values in parentheses are t-values.

#### The moderating effect was tested by grouping the median of internal control quality as the boundary

In order to further verify the internal control in government intervention and regulation of enterprise technology innovation, in this study, 13,159 study samples were divided into 6,580 low-quality internal control quality group data and 6,579 high-quality internal control quality group data, according to the boundary of median internal control quality. The government intervention in enterprise technological innovation in different control groups shows different regression results. By observing the regression equation coefficient and the significance of the model in the two groups, the effect of internal control on the influence mechanism of the two groups is judged.

Table 6 shows that in the low internal control quality group, the coefficient of government intervention variable and enterprises-based technological innovation is 0.28, and there is a positive correlation at a 1% level, indicating that the degree of government intervention can significantly hinder enterprise technological innovation. In the high-quality internal control group, the government intervention variable and enterprise technological innovation coefficient are 0.219. It is positively correlated at 1%, which is the same conclusion as the low internal control group.

**Table 6:** Testing moderating effect by groups.

	**A low-quality internal**	**The high-quality internal**
	**control group**	**control group**
**Variable**	**RD**	**RD**
Market	0.280^***^	0.219^***^
	(13.02)	(10.94)
Size	−0.039	−0.197^***^
	(−1.03)	(−6.31)
Lev	−0.042^***^	−0.036^***^
	(−17.18)	(−14.87)
Roa	0.007	0.024^***^
	(0.67)	(2.93)
Growth	−0.001	0.000
	(−0.82)	(0.50)
Fhold	−0.025^***^	−0.025^***^
	(−9.33)	(−10.60)
Age	−0.081^***^	−0.081^***^
	(−10.68)	(−11.86)
_cons	6.493^***^	9.775^***^
	(7.94)	(15.05)
*R* ^2^	0.136	0.166
*N*	6,580	6,579

*, **, and *** were significant at 0.1, 0.05 and 0.01 levels respectively. The values in parentheses are t-values.

In order to test whether there are differences in inter-group coefficients, Fischer combined test was conducted on the two groups of data in this article, and the results showed that the empirical *P*-value was 0.017 (see Table 7), indicating that there were significant differences in inter-group coefficients. By comparing the inter-group coefficients, it is found that in the low internal control quality group, the government intervention variables on enterprise technological innovation prove a weak promotion effect. In other words, in the low-quality internal control group, the degree of government intervention has a more substantial inhibition effect on enterprise technological innovation, indicating that the addition of internal control can strengthen the inhibition effect of government intervention on enterprise technological innovation.

**Table 7:** Fisher combination test results.

**Variables**	**b0-b1**	**Freq**	***p*** **-value**
Market	0.060	17	0.017
Size	0.158	0	0.000
Lev	−0.006	955	0.045
ROA	−0.017	855	0.145
Growth	−0.001	829	0.171
Fhold	0.000	554	0.446
Age	0.000	520	0.480
_cons	−3.282	1,000	0.000
Ho:b0(c.Market) = b1(c.Market)			
Observed difference = 0.060			
Empirical *p*-value = 0.017			

#### Utilization of the relationship between the government and the market for substitution of the market-oriented total score

Government intervention serves as the core variable of this article. In order to avoid the singleness of government intervention, the variables of government intervention are measured in this article by the relationship between government and the market, and the robustness of regression results is tested. The results are shown in Table 8. The multiple regression results are consistent with the above findings and consistent with the hypothesis of this article, which verifies the robustness of the research conclusions of this article.

**Table 8:** Regression results by substituting independent variables.

	**Model (1)**	**Model (2)**	**Model (3)**
Market	0.118^***^		0.116^***^
	(4.04)		(4.01)
IC		−0.061^*^	−0.072^**^
		(−2.47)	(−2.88)
Market ^*^ IC			−0.0578^**^
			(−2.74)
Size	−0.039	−0.033	−0.030
	(−0.71)	(−0.60)	(−0.55)
Lev	−0.020^***^	−0.020^***^	−0.019^***^
	(−8.20)	(−8.23)	(−8.18)
ROA	−0.001	0.002	0.002
	(−0.17)	(0.23)	(0.23)
Growth	−0.003^***^	−0.003^***^	−0.003^***^
	(−5.60)	(−5.37)	(−5.39)
Fhold	−0.006^*^	−0.007^*^	−0.007^*^
	(−2.25)	(−2.12)	(−2.20)
Age	−0.051^***^	−0.053^***^	−0.052^***^
	(−5.47)	(−5.64)	(−5.56)
Industry	Control	Control	Control
Year	Control	Control	Control
_cons	2.155	3.043^**^	2.440^*^
	(1.83)	(2.63)	(2.06)
*N*	12,178	12,178	12,178
*R* ^2^	0.157	0.157	0.158

*, **, and *** were significant at 0.1, 0.05 and 0.01 levels respectively. The values in parentheses are t-values.

## Conclusions and recommendations

Based on the A-share listed companies in Shanghai and Shenzhen stock markets from 2008 to 2018, the relationship between government intervention, internal control quality, and enterprise technological innovation is discussed in this article, enriching the existing literature. Research indicates that: (1) a Market economy with government intervention will restrain the technological innovation of enterprises. Government intervention provides enterprises with political resources and other helping hands, enabling enterprises to maintain regular operations by rent-seeking and other means. Therefore, high-risk and uncertain technological innovation activities will not be adopted to improve enterprise benefits. Based on the pressure of political indicators, enterprises focus on the realization of short-term goals and ignore the development of long-term strategies, thus inhibiting the technological innovation of enterprises. (2) The high level of internal control inhibits the technological innovation activities of enterprises. In order to strictly follow the rules and work in a fixed mode, the sense of constraint will significantly reduce the possibility of an employee in innovation. Innovation projects of enterprises may also miss innovation opportunities due to the lengthy approval cycle, thus affecting the innovation activities of enterprises. (3) The moderating effect of internal control on the impact of government intervention on enterprise technological innovation. The high quality of internal control fails to bring the advantage of system resources to enterprises; instead, it restricts the technological innovation of enterprises. When enterprises are under the government's excessive intervention and control, enterprises will fall into bondage. The high-quality internal control of enterprises will bring constraints to enterprises again and seriously inhibit technological innovation.

### Research-based recommendations

#### Be clear of government positioning and accelerate the marketization process

Based on the research conclusions, this article provides some instructive recommendations:

What kind of technological innovation should be decided by the enterprises, and the government should clarify its position, and government should not pose excessive intervention and control on the operational decisions of enterprises. The government should streamline administration and delegate power, simplify administrative approval procedures and reduce the possibility of rent-seeking by enterprises. At the same time, the government should correctly understand and handle the relationship with the market, play a critical role in allocating resources, and accelerate the marketization process to release the vitality of enterprises' technological innovation development. The improvement of marketization is conducive to grasping more opportunities for enterprises to promote innovative projects and then increasing enterprises' investment in technological innovation activities.

#### Establishing and improving the incentive mechanism for senior managers of enterprises

The principal-agent problem is a common phenomenon in enterprises. Solving the principal-agent problem has become a key factor for the long-term development of enterprises. When the performance evaluation index of enterprise management is preferably made to achieve short-term benefits, the manager will be more likely to ignore investment in R&D innovation for their interests, which hinders the long-term development of enterprises. Enterprises should establish and improve the incentive mechanism of management and strike a balance between short-term benefits and long-term development. Besides, we should increase evaluation indicators such as technological innovation and improve management's attention to technological innovation, thus improving the level of technological innovation, which is conducive to realizing the enterprise's long-term strategy.

#### Top priority to the cost-benefit principle of internal control to strengthen the flexibility of internal control

The design of the internal control system is made with the requirements of considering both efficiency and effect for sound implementation. We should not deviate from the original intention of internal control due to a tedious and all-inclusive internal control system, that is, to improve the operating efficiency and benefit of enterprises. Enterprises should follow the principle of cost and benefit and balance the relationship between the two to avoid a cumbersome approval process, which lacks flexibility and is hard to implement. A better understanding of the role of internal control in enterprises is conducive to the long-term development of enterprises.

## Data availability statement

The raw data supporting the conclusions of this article will be made available by the authors, without undue reservation.

## Ethics statement

Ethical review and approval was not required for the study on human participants in accordance with the local legislation and institutional requirements. Written informed consent for participation was not required for this study in accordance with the national legislation and the institutional requirements.

## Author contributions

SYe and SYi proposed the research idea, analyzed the results, and write the manuscript. SF and QY carried out the methodology and extensively edited the manuscript. All authors contributed to the article and approved the submitted version.

## Funding

This work was supported by Jilin University Clean Government Think Tank Innovation Team Project (2017LZZK008).

## Conflict of interest

The authors declare that the research was conducted in the absence of any commercial or financial relationships that could be construed as a potential conflict of interest.

## Publisher's note

All claims expressed in this article are solely those of the authors and do not necessarily represent those of their affiliated organizations, or those of the publisher, the editors and the reviewers. Any product that may be evaluated in this article, or claim that may be made by its manufacturer, is not guaranteed or endorsed by the publisher.
